# *E. coli*-Derived L-Asparaginase Retains Enzymatic and Cytotoxic Activity *In Vitro* for Canine and Feline Lymphoma after Cold Storage

**DOI:** 10.1155/2013/786162

**Published:** 2013-04-28

**Authors:** Jackie M. Wypij, Holly C. Pondenis

**Affiliations:** Department of Veterinary Clinical Medicine, University of Illinois at Urbana-Champaign, Hazelwood Drive, Urbana, IL 61802, USA

## Abstract

*Background*. L-asparaginase is effective in treating canine and feline lymphoma, however chemotherapy poses a significant financial cost to veterinary clients, limiting therapy for many pets. Single dose vials result in significant drug wastage, and drug shortages limit consistent availability for pets. *Hypothesis*. *E. coli*-derived asparaginase retains enzymatic and antineoplastic activity in canine and feline lymphoma cells after cold storage. *Methods*. *E. coli*-derived asparaginase was cold-stored: refrigeration (7–14 days) and freezing (14 days–six months, one to three freeze/thaw cycles). Enzymatic activity of asparaginase was measured via a modified asparagine assay. Effects of cold-stored asparaginase on cell proliferation and cytotoxicity were measured in feline (MYA-1, F1B) and canine (17–71, OSW) lymphoma cells. *Results*. Cold-stored *E. coli*-derived asparaginase retains antineoplastic activity in all four cell lines tested. Cold-stored *E. coli*-derived L-asparaginase depletes asparagine and retains enzymatic activity. Duration of refrigeration, duration of freezing, and number of freeze-thaw cycles have minimal effect on asparaginase enzyme activity. *Conclusions and Clinical Importance*. This study establishes a scientific basis for long-term cold storage of reconstituted *E. coli*-derived asparaginase that may result in better utilization of limited drug resources and improve financial feasibility of *E. coli*-derived asparaginase as a therapeutic option for pets with lymphoma.

## 1. Introduction

Lymphoma is the most common hematopoietic cancer affecting dogs and cats, accounting for approximately 25–30% of canine and feline cancer [1–5]. Chemotherapy treatment poses a significant financial cost to veterinary clients, limiting therapy for many pets with lymphoma. L-asparaginase (ASNase) is an effective chemotherapeutic routinely administered for canine [6, 7] and feline lymphomas [8–10]. *Escherichia coli*-derived L-asparaginase is available as a preservative-free lyophilized powder in single-use vials of 10,000 units/vial for injection. Manufacturer recommendations include storage of reconstituted solution at 2–8°C with discard after eight hours [11]. Given a standard veterinary dosage of 400 IU/kg to 10,000 IU/m^2^, a single vial of *E. coli*-derived ASNase may contain adequate drug to treat more than one small-sized patient. However, regardless of patient size there remains a fixed cost to the veterinarian and pet owner per single vial resulting in much of each drug vial being discarded. This creates significant drug wastage and added owner expense. These issues may be further compounded by limited availability during periods of drug shortage.

One potential method to overcome added drug cost and availability concerns is long-term cold storage of reconstituted drug. Many proteins retain enzymatic activity after cold storage and freeze-thaw conditions. A form of ASNase derived from *Erwinia carotovora* loses activity after freezing [12]; however an *E. coli* form retains at least structural stability after freeze-thaw [13]. To the authors' knowledge, effects of long-term cold storage on *E. coli*-derived ASNase activity have not been documented. We hypothesize that *E. coli*-derived ASNase retains enzymatic and antineoplastic activity after refrigeration or freezing. The goals of this study were to establish a scientific basis for long-term cold storage of reconstituted *E. coli*-derived ASNase to facilitate multiple doses in small animal patients with lymphoma. This may result in better utilization of limited drug resources and improve financial feasibility of *E. coli*-derived ASNase as a therapeutic option for pets with lymphoma.

## 2. Methods

### 2.1. Cell Culture and Reagents

Feline lymphoma cell line F1B and feline T lymphoblast cell line MYA-1 were purchased commercially from American Tissue Culture Collection (Manassas, VA, USA). Canine lymphoma cell lines 17–71 and OSW were provided by Dr. William Kisseberth and Dr. Steven Suter, respectively. All cell lines were grown in a humidified 5% CO_2_ environment maintained at 37°C in media containing 1% penicillin-streptomycin and passaged twice weekly. F1B cells were cultured in DMEM with 10% fetal bovine serum (FBS). For the asparagine assay only, F1B cells were supplemented at standard concentration of 10 ml/L media with nonessential amino acids (Gibco MEM Nonessential Amino Acids Solution, 100x, Invitrogen Life Technologies, Grand Island, NY, USA). MYA-1 cells were cultured in RPMI 1640 medium with 2 mM L-glutamine adjusted to contain 1.5 g/L sodium bicarbonate, 4.5 g/L glucose, 10 mM HEPES, and 1.0 mM sodium pyruvate and supplemented with 0.05 mM 2-mercaptoethanol, 100 units/mL recombinant human IL-2, and 10% fetal bovine serum. OSW and 17–71 canine lymphoma cells were maintained in RPMI medium with 10% FBS and 1% L-glutamine. *E. coli*-derived L-asparaginase (Elspar, Lundbeck, Deerfield, IL, USA) and L-asparagine (Sigma-Aldrich, St. Louis, MO, USA) were purchased. 

### 2.2. Cold Storage Conditions for *E. coli*-Derived L-Asparaginase

Each vial of lyophilized *E. coli*-derived ASNase was reconstituted aseptically as for clinical use per package instructions using 5 mL sterile water for injection per 10,000 unit vial with a final concentration of 2000 IU/mL. All subsequent experiments were performed with aseptic technique with microscopic monitoring for bacterial contamination, which was not observed. Aliquots were placed in cold storage under the following conditions: refrigeration (4°C/37°F, standard residential refrigerator temperature) for 7 or 14 days or freezing (−20°C/−4°F, standard residential freezer temperature) for 14 days or six months. Additional drug aliquots frozen for 14 days were subjected to from one to three freeze-thaw cycles per 14-day period.

### 2.3. Cell Proliferation and Cytotoxicity

All cell lines were seeded in 96-well plates at a density of 5 × 10^3^ cells/well and incubated in media with or without cold-stored *E. coli*-derived ASNase. Following 24 hr of drug exposure at 10 IU/mL [14], cell proliferation and cytotoxicity were assessed with commercial colorimetric and fluorometric assays, respectively (Aqueous One MTS and Multitox Fluor Multiplex, Promega, Madison, WI, USA), using manufacturer's recommended protocol. In brief, the cell proliferation assay is a colorimetric tetrazolium salt assay that measures cell viability. The cytotoxicity assay simultaneously measures the relative number of live and dead cells in cell populations to give ratiometric, inversely correlated measures independent of cell number. Live-cell protease activity is restricted to intact viable cells measured by a fluorogenic, cell-permeant peptide substrate (glycyl-phenylalanyl-amino fluorocoumarin (GF-AFC)). A second fluorogenic cell-impermeant peptide substrate (bis-alanyl-alanyl-phenylalanyl-rhodamine 110 (bis-AAF-R110)) is used to measure dead-cell protease activity. For each cell line results were averaged over four replicate experiments.

### 2.4. Asparagine and Asparaginase Enzyme Activity Assay

A commercial asparagine assay (rapid L-asparagine/L-glutamine/ammonia assay, Megazyme International Ireland, Co. Wicklow, Ireland) was used to quantify asparagine levels in a representative cell line (F1B). The enzyme asparaginase hydrolyzes L-asparagine into L-aspartate and ammonium ions; subsequent ammonium ion concentration measured colorimetrically at 340 nm is stoichiometric with L-asparagine concentration. The assay is specific for L-asparagine; the D-isomer does not react. Assay results are linear at 0.005–0.5 g/L of asparagine. Asparagine is considered a nonessential amino acid because, while noncancerous cells contain asparagine synthetase, malignant lymphocytes often lack this ability and are dependent on extracellular asparagine. As a representative cell line, F1B feline lymphoma cells were seeded in a 96-well plate at a density of 5 × 10^3^ cells/well overnight. Cells were incubated in media supplemented with nonessential amino acids (including L-asparagine) with or without *E. coli*-derived ASNase stored under the described conditions. Following 24 hr of drug exposure at 10 IU/mL [14], supernatant L-asparagine levels were assessed using manufacturer's recommended microplate format protocol. Results were averaged over four replicate experiments.

The assay was then modified to quantify ASNase enzyme activity. Two experiments were designed to validate assay modification via substitution of freshly prepared experimental *E. coli*-derived ASNase for the standard commercial assay. In the first part to validate equivalence of ASNase enzyme activity, a standard curve of eight serial dilutions of known asparagine concentrations (0.01–0.45 *μ*g/*μ*L) was prepared. Results obtained with substituted *E. coli*-derived ASNase were compared with results obtained with equivalent concentrations of the standard commercial assay ASNase. Results were averaged over four replicate experiments. In the second part to validate sensitivity to detect loss of ASNase enzyme activity, a known concentration of asparagine (0.1 *μ*g/*μ*L) was prepared. Results obtained with serially diluted substituted *E. coli*-derived ASNase (to 1 : 1000 dilution) were compared with serially diluted standard commercial assay ASNase (to 1 : 1000 dilution). Results were averaged over four replicate experiments. 

The modified assay was subsequently used to assess effects of cold storage on *E. coli*-derived ASNase enzyme activity. A known concentration of asparagine (0.1 *μ*g/*μ*L) was analyzed with the modified assay using cold-stored *E. coli*-derived ASNase in comparison to standard commercial assay as control. In this experiment, serial dilutions (to 1 : 1000 dilution) were used to concurrently compare loss of enzyme activity. Results were averaged over three replicate experiments.

### 2.5. Statistical Analysis

Commercial software programs (GraphPad InStat 3 and GraphPad Prism 5, San Diego, CA, USA) were used. Data were tested for normality using D'Agostino and Pearson omnibus K2 normality test. Parametric tests were used for normally distributed data which was expressed as mean ± standard deviation. Comparison of cold storage treatment on supernatant asparagine levels was performed with one-way ANOVA with Tukey posttest. Comparison of cold-stored drug treatment effects in four cell lines on cell proliferation and cytotoxicity was performed as a two-way ANOVA with Bonferroni posttest. Comparison of enzyme equivalence between assay types (standard versus modified assay) was performed as Pearson correlation with 95% confidence interval and coefficient of determination and as linear regression with 95% confidence interval and goodness of fit. Comparison of loss of enzymatic activity via dilution and assay type (standard versus modified assay) was performed as a two-way ANOVA with Bonferroni posttest. Comparison of concurrent effects of dilution and cold storage treatment on ASNase enzymatic activity was performed as a two-way ANOVA with Bonferroni posttest. Significance was set at *P* < 0.05 for all experiments.

## 3. Results

### 3.1. *E. coli*-Derived ASNase Retains Antineoplastic Activity

**Figure 1:**
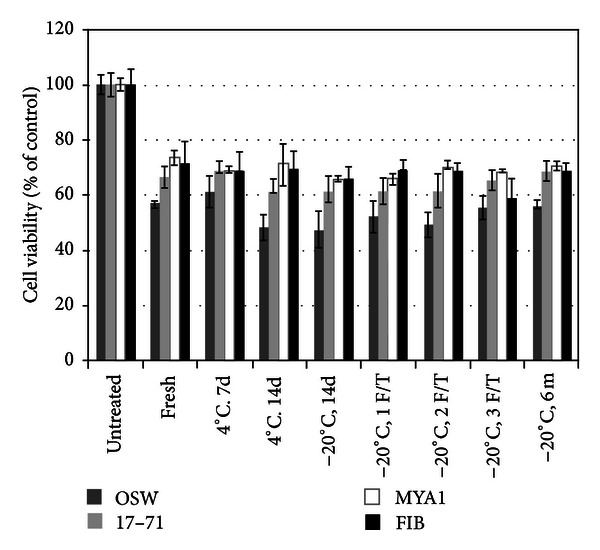
ASNase reduces cell proliferation *in vitro*. All cold-stored ASNase treatment groups decrease cell proliferation, *P* < 0.05. Cell lines demonstrate variable response to ASNase effects.

ASNase is used effectively in a clinical setting, and from morphological appearance of lymph node after treatment in a dog, appears to cause induction of apoptosis [15]. The *in vitro* effect of ASNase on canine and feline lymphomas cells has not previously been demonstrated. In an effort to quantify antineoplastic effects of cold-stored *E. coli*-derived ASNase in these cell types, we evaluated both effect of viability and cytotoxicity. A dose of 10 U/mL was used as previously described [15]. Although there is a linear correlation between *in vivo* and *in vitro* leukemia cell killing by ASNase, there does not appear to be a linear dose-response curve [16], and thus a single dose of 10 U/mL was used in this study. In all four cell lines tested, *E. coli*-derived ASNase decreased *in vitro* cell proliferation by approximately 25–50% (Figure 1). Results are significant for all cold-stored treatment groups compared to untreated media control cells (*P* value between *P* < 0.05 and *P* < 0.001). As previously reported [17], cell lines exhibit variable sensitivity to ASNase, with OSW cells exhibiting the greatest response (Figure 1).

**Figure 2:**
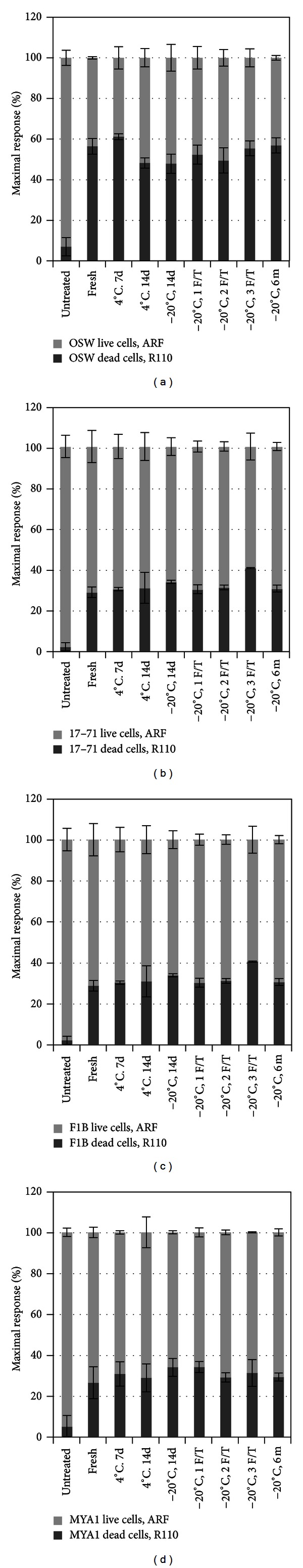
ASNase increases cytotoxicity *in vitro*. All cold-stored asparaginase treatment groups increase cell cytotoxicity, *P* < 0.05. (a) OSW canine cells, (b) 17–71 canine cells, (c) F1B feline cells, and (d) MYA1 feline cells. Cell lines demonstrate variable response to ASNase effects.

In all four cell lines tested, *E. coli*-derived ASNase also shifted the live-cell : dead-cell ratio confirming cytotoxic effect (Figure 2) with cell-kill rates of 27–61%. Results are significant for all cold-stored treatment groups compared to untreated media control cells (*P* value between *P* < 0.05 and *P* < 0.001). Similar to results of proliferation, cell lines exhibit variable cytotoxicity from ASNase, with OSW cells exhibiting the greatest response (Figure 2). These results of decreased cell proliferation and increased cytotoxicity demonstrate that *E. coli*-derived ASNase retains antineoplastic activity for lymphoma cells after cold storage. As previously reported for human and murine lymphomas [15–18], *E. coli*-derived ASNase appears to have direct cytotoxic effects on malignant canine and feline lymphocytes.

### 3.2. *E. coli*-Derived ASNase Depletes Asparagine *In Vitro* after Cold Storage

**Figure 3:**
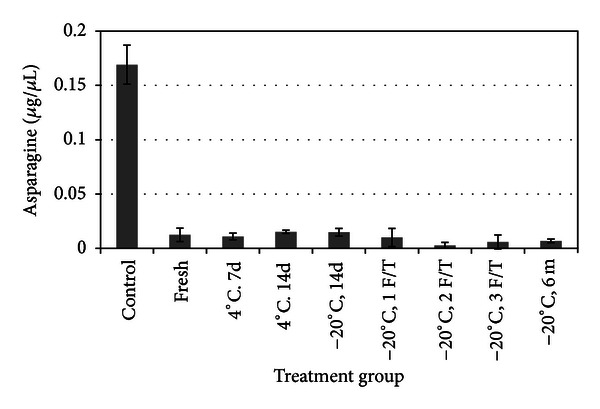
ASNase depletes supernatant asparagine *in vitro.* All cold-stored asparaginase treatment groups reduce asparagine levels compared to untreated cells, *P* < 0.0001. The effects of types of cold storage treatments are not significant (*P* > 0.05).

The mechanism of lymphoma cell kill appears to be hydrolysis and depletion of asparagine leading to apoptotic cell death [15, 18]. To test the relationship of ASNase hydrolysis of asparagine, F1B feline lymphoma cells were supplemented with nonessential amino acids (including L-asparagine) at standard concentration for mammalian cell culture (10 mL/L media). Supernatant collected after exposure to cold-stored *E. coli*-derived ASNase demonstrated significant decrease, with more than 90% asparagine depletion, in all treatment groups compared to untreated media control cell supernatant (Figure 3), *P* < 0.0001. There was no significant difference among treatment groups (Figure 3), *P* > 0.05. 

### 3.3. Modified Assay Detects ASNase Enzyme Activity

Prior to comparing the effects of cold storage, the commercial asparagine assay was modified to quantify ASNase activity. Validation of equivalence of standard commercial assay and modified assay to assess ASNase enzyme activity was performed using a colorimetric assay on an asparagine standard curve. Enzyme activity of the modified assay using *E. coli*-derived ASNase was linear and not statistically different from the standard commercial assay. Analysis of linear regression of ammonium ion concentration compared to the standard assay or modified assay was calculated with *r* ^2^ value of 0.9898, *P* < 0.0001 and *r* ^2^ value of 0.9816, *P* < 0.0001, respectively. Correlation of standard assay versus modified assay demonstrated a correlation coefficient *r* of 0.9981 (95% CI of 0.9891–0.9997) with a high coefficient of determination *r* ^2^ of 0.9962. Thus, the calculated straight line could explain more than 99% of the experimental data, demonstrating equivalence between the standard and modified assay. 

**Figure 4:**
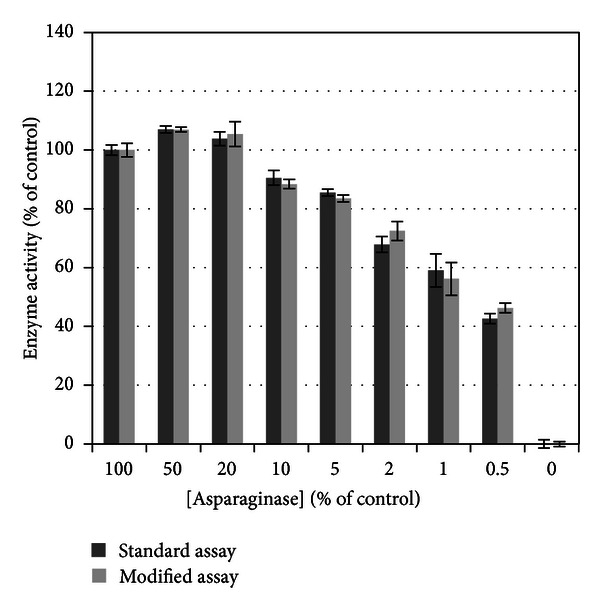
Loss of asparaginase enzyme activity. Asparaginase dilution has a significant effect on enzyme activity (*P* < 0.0001); effects of assay types (standard commercial versus modified) are not significant (*P* = 0.1059). The modified assay demonstrates loss of enzyme activity similar to that seen with the standard commercial assay.

Having validated the equivalence of the modified assay, there remained the concern that, in a setting of enzyme excess, loss of ASNase activity would be difficult to identify. Validation of the modified assay's sensitivity to detect loss of ASNase enzyme activity compared to the standard assay was performed. Serial dilution of ASNase in both standard and modified assays was performed. Enzyme activity was maintained near 100% of controls through a dilution of one to five (20% of initial ASNase concentration) and was attenuated with ASNase concentration ≤10% of control (1 : 10 dilution), *P* < 0.0001. There was no statistical difference between results obtained with standard commercial assay and modified assay, *P* = 0.1059 (Figure 4). 

Together, these results validate equivalence of assay modification and ability to detect loss of ASNase enzyme activity, supporting further use in evaluating ASNase enzyme activity in cold-stored drug. 

### 3.4. *E. coli*-Derived L-Asparaginase Retains Enzymatic Activity after Cold Storage

**Figure 5:**
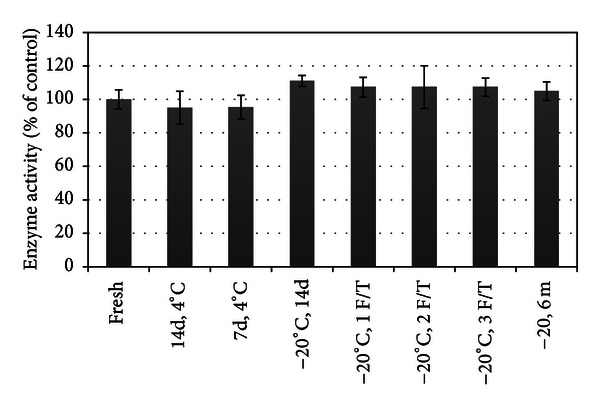
Effect of cold storage on asparaginase enzyme activity. There is no significant difference between types of cold storage on asparaginase activity, *P* = 0.0637.

**Figure 6:**
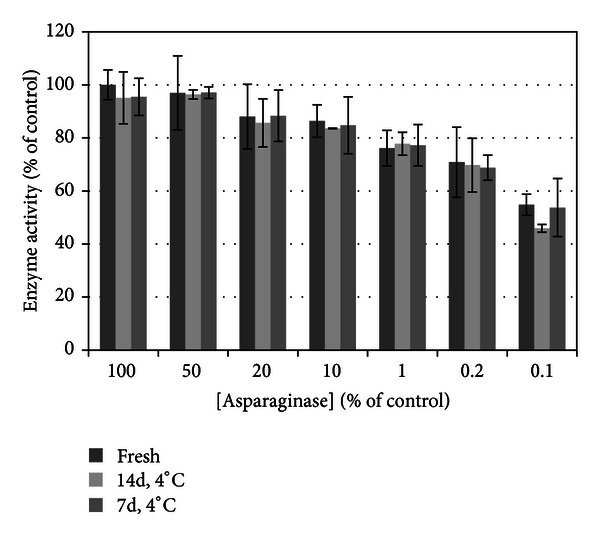
Effect of refrigeration on asparaginase enzyme activity. There is no significant difference in asparaginase activity between refrigeration and freshly reconstituted drug or with duration of refrigeration, *P* > 0.05. As expected, asparaginase concentration has a significant effect on enzyme activity (*P* < 0.0001), but no difference between cold storage types at any concentration tested. This verifies that refrigerated drug retains enzyme activity at 7 and 14 days similar to freshly prepared drug.

**Figure 7:**
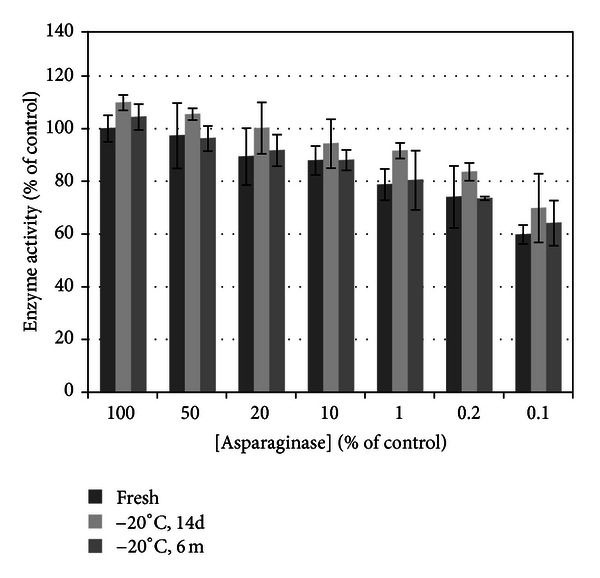
Effect of freezing on asparaginase enzyme activity. Duration of freezing accounts for a minor portion of the statistical difference between cold storage groups. Post hoc tests did not identify a significant difference between either freezing durations compared to control. This verifies that frozen stocks retained enzyme activity up to 6 months similar to freshly prepared drug.

**Figure 8:**
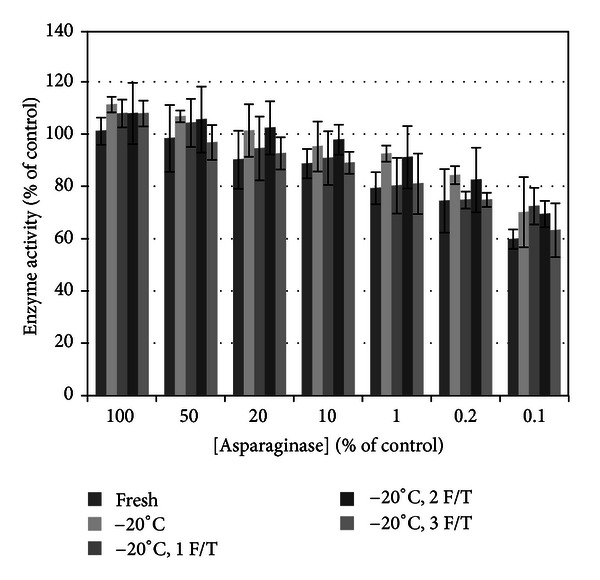
Effect of freeze/thaw on asparaginase enzyme activity. The number of freeze/thaw cycles accounts for a minor portion of the statistical variance between cold storage groups. Post hoc tests did not identify a significant difference between any cold storage type compared to control. This verifies that frozen stocks retained enzyme activity with freeze-thaw cycles similar to freshly prepared drug.

The modified assay as detailed previously was subsequently used to detect ASNase activity in cold-stored ASNase treatment groups to quantify effects of cold storage. Enzyme activity in all treatment groups approximated 100% of control, and there was no statistical difference based on type of cold storage (Figure 5), *P* = 0.0637. Given that ASNase enzyme activity is in excess, with no loss until ASNase concentration is ≤10% of control, dilutions were performed to verify loss of enzyme function (Figures 6–8). As expected, ASNase concentration (dilution factor) was considered significant for enzyme activity in all experiments (*P* < 0.0001). 

Effect of refrigeration on enzyme activity (Figure 6) was not significantly different between type and duration of cold storage (*P* = 0.5419). This demonstrates that refrigerated *E. coli*-derived ASNase retains similar enzyme activity to freshly prepared drug. 

Effect of duration of freezing on enzyme activity (Figure 7) was significant (*P* < 0.05) with duration of freezing accounting for a minor 8.12% of the total variance compared to ASNase dilution accounting for 73.99% of the variance. Bonferroni posttests demonstrated no significant differences, with both 14-day and six-month duration of freezing being statistically similar to freshly prepared drug (*P* > 0.05). The mean enzyme activity of the frozen samples was higher than freshly prepared control in >75%. This demonstrates that frozen *E. coli*-derived ASNase does not lose enzyme activity compared to freshly prepared drug. 

Effect of freeze/thaw cycles on enzyme activity (Figure 8) was significant (*P* < 0.05) with freeze/thaw cycles accounting for a minor 6.44% of the total variance compared to ASNase dilution accounting for 70.97% of the variance. Bonferroni posttests demonstrated no significant differences with one to three freeze/thaw cycles being statistically similar to freshly prepared drug (*P* > 0.05). The mean enzyme activity of the frozen samples was higher than freshly prepared control in >75%. This demonstrates that *E. coli*-derived ASNase subjected to freeze/thaw cycles does not lose enzyme activity compared to freshly prepared drug. 

## 4. Discussion 

*E. coli*-derived ASNase is known to retain activity for two days at room temperature and seven days when refrigerated [19, 20]. A related form of ASNase (derived from *Erwinia carotovora*) was shown to lose activity after freezing [12]. Our study demonstrates that refrigerated and frozen *E. coli*-derived ASNase retains antineoplastic activity as evidenced by reduction in cell proliferation and induction of cytotoxicity in feline and canine lymphomas cells *in vitro*. In addition, cold-stored ASNase depletes asparagine in cell culture. We were able to develop a modified assay of enzyme activity that demonstrated that cold-stored *E. coli*-derived ASNase has similar enzyme activity to freshly prepared drug. Duration of freezing and multiple freeze-thaw cycles had minimal effect and in fact, in >75% of the testing scenarios, had increased activity compared to freshly prepared drug. Although it is unlikely that enzyme activity increases with freezing, this may be a result of minor dehydration of sample resulting in concentration of the resultant drug stock. 

## 5. Conclusions 

Results of this study suggest that *E. coli*-derived L-asparaginase retains drug efficacy *in vitro* following cold storage, including refrigeration for up to 14 days, freezing for up to six months, and repeated freeze-thaw cycles. This establishes a scientific basis for long-term cold storage of reconstituted *E. coli*-derived L-asparaginase to facilitate multiple doses in small animal patients with lymphoma. This practical information may be clinically useful and result in better utilization of limited drug resources and improve financial feasibility of *E. coli*-derived L-asparaginase as a therapeutic option for pets with lymphoma. Limitations of this study include its *in vitro* nature and limited length of time of cold storage. Unresolved issues include the extra-label use of a drug packaged for single use in this fashion as well as concerns for sterility and patient safety when using a single-dose, preservative-free solution for multiple doses in treating client-owned pet animals with lymphoma.
